# Soil Properties Interacting With Microbial Metagenome in Decreasing CH_4_ Emission From Seasonally Flooded Marshland Following Different Stages of Afforestation

**DOI:** 10.3389/fmicb.2022.830019

**Published:** 2022-02-23

**Authors:** Qian Zhang, Jie Tang, Roey Angel, Dong Wang, Xingyi Hu, Shenghua Gao, Lei Zhang, Yuxi Tang, Xudong Zhang, Roger T. Koide, Haishui Yang, Qixiang Sun

**Affiliations:** ^1^Research Institute of Forestry, Chinese Academy of Forestry, Beijing, China; ^2^Hunan Academy of Forestry, Changsha, China; ^3^Soil and Water Research Infrastructure and Institute of Soil Biology, Biology Centre, Czech Academy of Sciences (CAS), České Budějovice, Czechia; ^4^Institute of Forest Ecology, Environment and Nature Conservation, Chinese Academy of Forestry, Beijing, China; ^5^Hubei Academy of Forestry, Wuhan, China; ^6^Department of Biology, Brigham Young University, Provo, UT, United States; ^7^College of Agriculture, Nanjing Agricultural University, Nanjing, China

**Keywords:** CH_4_ flux, soil metagenome, methanogens, methanotrophs, soil particle size composition

## Abstract

Wetlands are the largest natural source of terrestrial CH_4_ emissions. Afforestation can enhance soil CH_4_ oxidation and decrease methanogenesis, yet the driving mechanisms leading to these effects remain unclear. We analyzed the structures of communities of methanogenic and methanotrophic microbes, quantification of *mcr*A and *pmo*A genes, the soil microbial metagenome, soil properties and CH_4_ fluxes in afforested and non-afforested areas in the marshland of the Yangtze River. Compared to the non-afforested land use types, net CH_4_ emission decreased from bare land, natural vegetation and 5-year forest plantation and transitioned to net CH_4_ sinks in the 10- and 20-year forest plantations. Both abundances of *mcr*A and *pmo*A genes decreased significantly with increasing plantation age. By combining random forest analysis and structural equation modeling, our results provide evidence for an important role of the abundance of functional genes related to methane production in explaining the net CH_4_ flux in this ecosystem. The structures of methanogenic and methanotrophic microbial communities were of lower importance as explanatory factors than functional genes in terms of *in situ* CH_4_ flux. We also found a substantial interaction between functional genes and soil properties in the control of CH_4_ flux, particularly soil particle size. Our study provides empirical evidence that microbial community function has more explanatory power than taxonomic microbial community structure with respect to *in situ* CH_4_ fluxes. This suggests that focusing on gene abundances obtained, e.g., through metagenomics or quantitative/digital PCR could be more effective than community profiling in predicting CH_4_ fluxes, and such data should be considered for ecosystem modeling.

## Introduction

Globally, natural wetlands are the largest source of CH_4_ emissions, accounting for ∼62% of the (natural) CH_4_ budget (Nazaries et al., 2013a). Emitted CH_4_ is almost exclusively microbial in origin, and its magnitude is determined by the balance between methane production and oxidation (Conrad, 1996). Previous studies showed that both methanogenic and methanotrophic microbes can be influenced by a variety of soil variables including soil C/N ratio, pH, temperature and NH_4_^+^ concentration (Kolb, 2009; Bodelier, 2011; Yuan et al., 2016; Zu et al., 2016; Wen et al., 2017). Other soil properties, such as water and clay contents and level of compaction, directly affect CH_4_ fluxes by physically controlling the diffusive transport of CH_4_ in soils (Kolb, 2009). However, few studies have analyzed the relationships among CH_4_ fluxes, methanogenic and methanotrophic communities, and these relevant soil properties.

The effects of land-use change on the strengths of CH_4_ sources and sinks and methanogenic or methanotrophic microbial communities are well documented. Afforestation is frequently examined because forest soils represent a significant sink in the global CH_4_ budget (Dalal and Allen, 2008). The uptake of atmospheric CH_4_ by the soil is often reported to increase due to afforestation, which is accompanied by a shift in the microbial community structure from type I to type II methanotrophs (Singh et al., 2007, 2009; Dörr et al., 2010). Studies have also shown that increased CH_4_ sink strength following afforestation is associated with increased dominance of members of the USC α clade-type-II-related methanotrophs (Nazaries et al., 2011, 2013b; Täumer et al., 2021). However, these studies did not include an analysis of the methanogenic community, which may also have been altered by afforestation and could thus affect the net flux of CH_4_ flux independent of the methanotrophic community. Thus, the lack of studies that include analyses of both methanogenic and methanotrophic communities strongly limits our understanding of the mechanisms by which CH_4_ emissions are mitigated by afforestation.

Freshwater marshlands, natural freshwater wetlands, are transitional zones bordering rivers and islands. They frequently experience seasonal flooding and are prone to erosion. In China, a heterogeneous marshland area of >900,000 ha is distributed in the middle and lower reaches of the Yangtze River. This area experiences a subtropical monsoon climate and usually has abundant seasonal rainfall. There is a rainy season (April–July) and a dry season (August–March). An ecologically fragile area, severe land loss and degradation occur due to continuing population growth, extensive land reclamation, and pollution (Jiang et al., 2015). Since the early 1980s, afforestation with poplar trees has been used to restore some of the degraded marshland along the Yangtze (Zhou et al., 2010). Our previous studies showed that poplar afforestation of marshland alters soil structure, increases soil organic matter content, and reduces CH_4_ emission (Zhou et al., 2010; Gao et al., 2013). However, the mechanism by which this is accomplished remains unknown. In particular, we do not know how afforestation has affected methanogenic and methanotrophic communities, nor do we know how relevant functional genes respond to poplar afforestation.

Methanogenic communities have been characterized using the 16S ribosomal RNA (rRNA) gene or using specific functional genes such as the α-subunit of the methyl-coenzyme M reductase (*mcr*A), which is involved in the final step of methanogenesis (Luton et al., 2002; Friedrich, 2005). Methanotrophs are usually dominated by methane-oxidizing bacteria (MOB), though anaerobic methane-oxidizing (ANME) archaea sometimes also make a small contribution to methane oxidization (Ettwig et al., 2016). For MOB, the *pmo*A gene, encoding the α-subunit of *p*MMO (the particulate form of methane monooxygenase), has a near-universal presence in both aerobic and nitrite-reducing bacterial methanotrophs and has been used as a biomarker for characterizing their communities and activities (Freitag and Prosser, 2009; Seo et al., 2013; Lee et al., 2014; Tate, 2015). Other than targeting a specific molecular biomarker, metagenomics approaches with genome shotgun sequencing open the opportunity to describe the diversity profile functional pathways governing key soil process like C, N and methane metabolism. However, metagenomics has only been used in a few studies of ecosystem restoration and succession. These include changes in functional genes between pre-agricultural tallgrass prairie and modern agricultural soils (Fierer et al., 2013), the impact of engineered soil formulations on microbial functions in restored mine sites (Kumaresan et al., 2017), and how plant-driven changes shape microbial communities during succession post agricultural abandonment (Cline and Zak, 2015). Soil microbial mediated biochemical pathways of methane production-oxidation and nitrogen metabolism were studied through metagenomics approach (Bhattacharyya et al., 2017). In this work, our goal was to expand our understanding of the changes due to afforestation in soil methane-cycling microbial communities and their function in marshland of the Yangtze River by using metagenomics together with 16S rRNA gene sequencing and quantification of specific marker genes. We used shotgun metagenomics to directly measure the changes in abundance of CH_4_ cycling functional genes, including methane production and methane oxidation. We hypothesized that (1) afforestation decreases the abundance of methanogens and methanogenesis functional genes but increases the abundance of methanotrophs and methane oxidation genes; (2) methane-related microbial communities, functional genes and net CH_4_ fluxes are influenced by changes in soil physical and chemical properties associated with afforestation; (3) methane related functional gene composition will be more powerful than taxonomic microbial community structure in explaining *in situ* CH_4_ flux.

## Materials and Methods

### Site Description

The field sampling was conducted in a freshwater marshland (28°59′–29°38′ N, 112°43′–113°15′ E) located in Junshan District, Hunan Province. The area of sampling is located at the west of the Yangtze River and northeast of Dongting Lake and possesses a typical subtropical humid climate, with mean annual precipitation of 1,417 mm and mean annual temperature of 16.4-17°C. It is characterized as a type of tidal soil developed from alluvial parent material, with organic matter of 2.43 g kg^–1^, total nitrogen of 1.26 g kg^–1^, available nitrogen of 112 mg kg^–1^ and pH of 7.9. This sampling site is annually immersed in a shallow layer of tidal water from May to August. The natural vegetation is dominated by *Cynodon dactylon* (L.) Pers. (Scutch grass), *Viola verecunda* A. Gray., *Polygonum flaccidum* Meisn. and *Clinopodium gracile* (Benth.) Kuntze, with vegetation cover of approximately 50%. In contrast, the vegetation cover is less than 1% in eroded areas of the marshland. To restore and prevent land degradation, poplar (*Populus deltoides* W. Bartram ex Marshall cv. ‘Lux’; Eastern cottonwood) plantations have been established in the past 30 years and were never fertilized.

### Soil Sampling

Five land types were selected within a 10 km × 10 km range in the outside-embankment marshland of the Yangtze River, including bare land (BA), natural vegetation (NV), and poplar plantations planted five years ago (PP5), ten years ago (PP10) and 20 years ago (PP20). Natural vegetation refers to the vegetation that develops in a natural state without artificial management. The forest plantations had a density of 318 individuals per ha and an average tree height of 17-20 m at sampling time. The tree height is similar at the sites for plantations of 5, 10, and 20 years. The popular trees grow very fast within the first 5 years after being planted when they can grow to a height of about 17 m (personal observation). After that the height only varies with a small increase of about 2–3 m. The afforested areas of the marshlands continue to be periodically flooded from end of May to August at the same time as the non-afforested areas. The understory plant growth in the plantations had a cover of <10%, which is caused by canopy closure and by the seasonal three-month-long immersion.

For each land type, three replicate sampling sites were selected, and one 10 m × 10 m sampling plot was set up within each sampling site (Supplementary Figure 1). The soil sampling was conducted at the end of August after three months‘ flooding. Five soil cores (40 cm deep and 4 cm inner diameter) were taken from each sampling plot. One sampling point was at the center of each plot, and the other four sampling points were located near each of the four corners of the plot at distances of 3 m to the border. The five soil cores at 0–10 cm, 10–20 cm, 20–30 cm, and 30–40 cm from each plot were first mixed thoroughly, and then an equal amounts of the mixed soil samples were taken and pooled again to form a composite sample, resulting in a total of 15 samples (5 land types × 3 replicates). Sampling was carried out immediately after the three-month flooding in August 2017. Only 1 kg of the pooled soil samples were sealed in plastic bags and refrigerated, immediately transported to the laboratory and sieved to 2 mm to remove root debris and stones. Soil samples were preserved at −80°C for subsequent molecular analysis. Sub-samples from each soil sample were air-dried for physicochemical analysis.

### DNA Extraction, PCR and 16S Amplicon Sequencing

Soil genomic DNA was extracted from 0.5 g fresh soil samples using the FastDNA SPIN Kit (MP Biomedicals, Santa Ana, CA, United States) following the manufacturer’s protocol. The extracted DNA was diluted to 10 ng μL^–1^ with the aid of a NanoDrop ND-1000 spectrophotometer (NanoDrop Technologies). The primer sets 343F/798R and A533F/A934R were used to amplify the bacterial and archaeal 16S rRNA gene, respectively (Table 1). PCR reactions contained 9 μL PCR water (Qiagen), 12.5 μL 2 × Ace Taq Master Mix (Vazyme Biotech), 0.5 μL each of the forward and reverse primers (10 μM each), with 2.0 μL (10 ng) of purified DNA from each sample as the template. Reactions were held at 95°C for 5 min to activate the polymerase, and then 28 cycles were performed at 95°C for 30 s, 55°C for 30 s, and 72°C for 30 s; and a final extension of 7 min at 72°C. Triplicate PCRs were conducted for each sample, and the three PCR products were pooled for high-throughput sequencing. After purification and quantification, 0.01 ug of PCR product from each sample was used for Illumina paired-end library preparation, cluster generation and sequenced on the Miseq Illumina PE 300 platform. Sequencing services were provided by (HanYu Laboratories, Shanghai, China). Raw sequence data are deposited in the BioProject database of the NCBI Short Read Archive under accession number PRJNA787026.

**TABLE 1:** Reads abundance of methanogenic genera across different land types from 16S amplicon sequencing. Data are mean reads abundance of samples per vegetation type (*n* = 3).

	BA	NV	PP5	PP10	PP20
*Methanobacterium*	219	77	22	47	15
*Methanobrevibacter*	0	0	1	0	0
*Methanothermobacter*	0	1	0	1	0
*Methanocella*	46	60	31	62	37
*Candidatus_Methanoregula*	11	56	8	94	17
*Methanolinea*	1	1	1	7	1
*Methanospirillum*	3	10	3	20	5
*Methanosaeta*	50	116	2	33	7
*Methanolobus*	0	0	0	0	1
*Methanosarcina*	53	175	40	147	31
*Methanomassiliicoccus*	9	17	11	4	22

*BA, bare land; NV, natural vegetation; PP5, 5-years old poplar plantation; PP10, 10-year old poplar plantation; PP20, 20-year old poplar plantation. Grayed cells indicate presence.*

### Bioinformatics of 16S Amplicon Data

The high throughput sequencing data were processed using QIIME 1.6.0 (Caporaso et al., 2010). The DNA reads were assigned to samples according to unique barcodes. The corresponding paired reads were merged if the overlap was 100% identical using FLASH (v 1.2.7)^1^. Quality filtering was done using QIIME, with the default settings for Illumina were (*r* = 3; *p* = 0.75 total read length; *q* = 3; *n* = 0) as recommended by Bokulich et al. (2013). Chimera sequences were removed with UCHIME (Version 4.2) implemented in QIIME (Edgar et al., 2011).

Operational taxonomic units (OTUs) were defined with 97% sequence similarity with Uparse^2^ (Edgar, 2013) after removal of putative chimera and singletons. The most abundant sequence was selected as the representative of each OTU. The taxonomy of each OTU was assigned through the RDP Classifier (version 2.2)^3^ (Sul et al., 2011) and trained on the Greengenes reference sequences^4^ (DeSantis et al., 2006). When OTUs were assigned to taxonomy, only OTUs annotated as bacteria or archaea were maintained. In addition, we also selected the methanogens and methanotrophs at the genus level for further analysis based on the taxonomic information. Two alpha diversity indices, observed species and chao1, were calculated.

### Quantitative PCR

Quantifications of soil methanogens were performed by quantitative PCR (qPCR) with primer sets mlas-mod–F/-mcrA-rev-R for the *mcr*A gene and A189f/mb661r for the *pmo*A gene. Because some studies reported that the primer set A189f-mb661r for *pmo*A could also amplify *amo*A, we did primer evaluation before qPCR. No clone sequenced from clone libraries generated by A189f-mb661r was identified as *amo*A. This was supported by a previous study which showed that sequences generated with primer set A189f-mb661r all grouped with the gammaproteobacterial *pmo*A sequences in a neighbor-joining phylogenetic tree (Redmond et al., 2010). The overall bacterial and archaeal abundance were also quantified by qPCR with the primer pairs Ba519F/Ba907R and Ar364F/Ar934R, respectively (Supplementary Table 1). PCR amplifications were carried out in triplicate on a CFX Connect Real-Time PCR Detection System (Bio-Rad) in a final volume of 10 μL containing 1 μL of gDNA, 0.4 μM of each respective primer, 5 μL of 2 × SG Green qPCR Mix (SinoGene). All assays also systematically included positive and negative controls. PCR amplification for bacterial and archa archeael eal 16S rDNA genes followed the procedure described in Angel et al. (2012). For *mcr*A and *pmo*A, the PCR thermocycling was initiated at 95°C for 3 min, followed by 40 cycles of 10 s at 95°C, 20 s at 58°C, and 10 s at 72°C. Fluorescence data were collected at the end of each cycle. To assess the specificity of amplification, a melting curve analysis was performed. Standard curves were created by plotting the cycle threshold (CT) values of the qPCRs performed on dilution series of plasmid DNA (10^4^-10^9^ copies/μl). The gene copy number in each analyzed sample was determined by comparing to Ct values/gene copy number of the standard curve.

### Metagenomic Analysis

DNA was fragmented to an average size of about 350 bp using Covaris M220 (Gene Company Limited, China) for paired-end library construction. A paired-end library was prepared using the TruSeq DNA Sample Prep Kit (Illumina, San Diego, CA, United States). Adapters containing the full complement of sequencing primer hybridization sites were ligated to the Blunt-end fragments. Paired-end sequencing was performed on Illumina HiSeq 4000 PE150 platform (Illumina Inc., San Diego, CA, United States) using HiSeq 3000/4000 PE Cluster Kit and HiSeq 3000/4000 SBS Kits according to manufacturer instructions. Raw sequence data are deposited in China National Genebank (CNGB) database under accession number CNP0002514.

Raw data were first processed by Trimmomatic 0.36 (Bolger et al., 2014) for adapter removal and moderate-quality trimming to obtain clean data for subsequent data analysis. Sequences containing N base and adapters or a low-quality value (Q value less than 20) were removed. De bruijn-graph-based assembler Megahit (v1.0.6) (Li et al., 2015) was employed to assemble short reads, with mapped ratio between 19.7 and 40.1% across all the samples. Open reading frames (ORFs) from each metagenomic sample were predicted using Prodigal (v2.60) (Hyatt et al., 2012). The predicted ORFs with lengths over 100 bp were retrieved and translated to amino acid sequences using the NCBI translation table.^5^ All sequences from gene sets with a 95% sequence identity (90% coverage) were clustered as the non-redundant gene catalog by the CD-HIT.^6^ Non-redundant sequences were mapped with Bowtie 1.1.2, and sam2counts 0.91 was used to convert mapping results to reference sequence counts, generating the gene table which could be used for functional annotation. Kyoto Encyclopedia for Genes and Genomes (KEGG)^7^ was used to analyze the functional aspects (Kanehisa and Goto, 2000). KOBAS 2.0 software was used for KEGG functional orthologs (KOs) annotation based on the non-redundant gene (Xie et al., 2011). Subsequently, all contig data were mapped against the KEGG pathway (Meyer et al., 2008) to identify tentative metabolic pathways for a specific function. To avoid the sequencing depth bias in comparative analysis, the total sequencing number was normalized to one million reads per sample. Genes relating to methanogenesis and methane oxidation were of particular interest and were selected for more detailed analysis. The pathway, ‘ko00680: Methane metabolism’, was considered as the core pathway related to methane production and consumption. The abundance of metagenomic reads assigned to a particular gene is used as a proxy for gene abundance in a sample.

### CH_4_ Flux Measurement

Data of methane flux was collected with a static dark chamber-FMA (fast methane analyzer) approach (Gao et al., 2013). The chamber was composed of three parts: a stainless steel base (60 cm × 60 cm × 12 cm), a joint chamber (60 cm × 60 cm × 120 cm) and a covering chamber (60 cm × 60 cm × 40 cm). The joint and covering chambers were made of tranparent plexiglass inert to CH_4_. The base was inserted into the soil at a depth of 10 cm. The covering chamber was equiped with two fans (diamer: 12 cm) to ensure that gas was mixed completely. The dark chamber was made through wrapping a layer of insulation with aluminum foil to minimize the internal temperature changes. The temperature in soils and chambers were measured with a digital thermometer. Gas samples (1.2 L) were collected at 2, 12, and 22 min after the covering chamber being capped, and stored in air-sampling bags (2 L) made of aluminum composite membrane. Gas sample collection was carried out with an interval of 6 h between the two adjacent collections. CH_4_ flux was then measure with LGR Fast Methane Analyzer (FMA) (Los Gatos Research, Ltd., San Joes, CA, United States). The CH_4_ flux was calculated as follows:


$$\text{F}=\frac{d\,c}{d\,t}*\frac{P}{P_{0}}*\frac{T_{0}}{T}*\frac{M}{22.4}*H$$

Here, F refers to the CH_4_ flux (ug m^–2^ h^–1^). *dc/dt* is the rate of CH_4_ accumulated in the chamber (PPBV CH_4_–C h^–1^). *P* is the atmospheric pressure (Pa) at the sampling sites. *P*_0_ is the standard atmospheric pressure (Pa). T is the air temperature (K) in the chamber at gas sampling. *T*_0_ is the air temperature (K) at the standard conditions. *M* is the molecular weight of CH_4_. *H* is the total height (cm) of chambers, including the joint and covering chambers.

### Soil Properties

Soil samples were assayed for particle composition, organic matter, total nitrogen, available nitrogen, total phosphorus, extractable phosphorus, total potassium, available potassium, pH and cation exchange capacity (CEC). Soil particle composition was determined using Mastersizer 2000 (Malvern Panalytical, United Kingdom). Organic matter was determined by the standard Walkley-Black potassium dichromate oxidation method (Nelson and Sommers, 1982). Total N concentrations were measured by the Kjeldahl procedure (2200 Kjeldahl Auto Distillation) and available N (AN) by the alkaline hydrolysis diffusion method (Lu, 1999). Total P and extractable P concentrations were measured by spectrophotometry (UV-1600 spectrophotometer, Beijing) (Lu, 1999). Total K and available K (AK) were determined by flame photometry (Lu, 1999). Soil CEC was measured by the ammonium acetate method at pH 7.0 (Lu, 1999). The value of soil pH was determined in 1:1 soil:water slurries with an acidometer (HANNA, Padua, Italy). Soil dry matter was determined by drying samples at 105 ± 2°C until it reached a constant mass as laid out in the Soil Quality ISO Section 3.1 (ISO 11465, 1993).

Oxygen (O_2_) concentrations were measured by gently pushing a Clark type glass microelectrode (500 μm tip, Unisense A/S Aarhus N, Denmark) into the soil at 20 cm depth. The microelectrode was positioned by micromanipulator, and the sensor current was measured with a picoammeter (PA2000, Unisense A/S). The microelectrode was calibrated with both air-saturated and oxygen-free N_2_-saturated water at the same temperature as the soil.

### Random Forest Analysis

To explore the role of soil properties, abundances of methanogens and methanotrophs, and vital functional genes in driving CH_4_ fluxes, we used a regression Random Forest (RF) analysis (Breiman, 2001) to identify the main predictors of CH_4_ flux. In our RF analyses, the soil particle composition, relative abundance of methanogens and methanotrophs at the genus level, quantification of *mcr*A and *pmo*A genes, and abundances of key functional genes revealed with metagenomics were included as predictors of CH_4_ flux. The relative abundance of methanogens and methanotrophs were calculated as follows: first, we selected the genera belonging to the known methanogens and methanotrophs; then using the abundance of these genera to calculate the relative abundance of methanogens with the total abundance of archaeal 16S high-throughput sequencing reads, as well as for methanotrophs with bacterial 16S. The response variable was the mean values of the four measurements of CH_4_ flux. These analyses were conducted using the RF package (Liaw and Wiener, 2002) for R. The significance of the model and the cross-validated *R*^2^ were assessed with 5,000 permutations of the response variable, using the A3 (Fortmann-Roe, 2015) R package. Similarly, the significance of the importance measures of each predictor on the response variable (enzymatic activities) was assessed by using the *rfPermute*^8^ package for R.

### Structural Equation Model

To identify drivers of CH_4_ flux, structural equation models (SEM) using Mantel R values as inputs were constructed in AMOS 20.0 (Arbuckle, 2011). We included variables identified to be important predictors in RF analysis to explore direct effects and their interactive effect of these variables. A maximum likelihood estimation method was used to compare the SEM models with observations. The goodness of fit was determined with the criterion of RMSEA (root mean squared error of approximation) < 0.05 and TLI (Tucker-Lewis Index) > 0.95 as suggested by Hu and Bentler (1999).

### Statistical Analysis

Anaerobic archaeal methanogens and aerobic bacterial methanotrophs were identified based on the taxonomic information at the genus level with high-throughput 16S amplicon sequencing (see the review of Nazaries et al., 2013a). Non-metric multidimensional scaling (NMDS) analysis was performed based on the relative abundance of each genus to illustrate differences in the structure of the methanogenic and methanotrophic communities among land types. Statistically significant difference was determined with the analysis of similarity (ANOSIM) for methanogenic or methanotrophic communities among different land types (Vegan package in R, Oksanen et al., 2013). Non-parametric Kruskal-Wallis test was performed to test the difference of each genus among land types (Agricolae package in R, de Mendiburu, 2012).

## Results

### Community Structure of Methanogens and Methanotrophs

**FIGURE 1:**
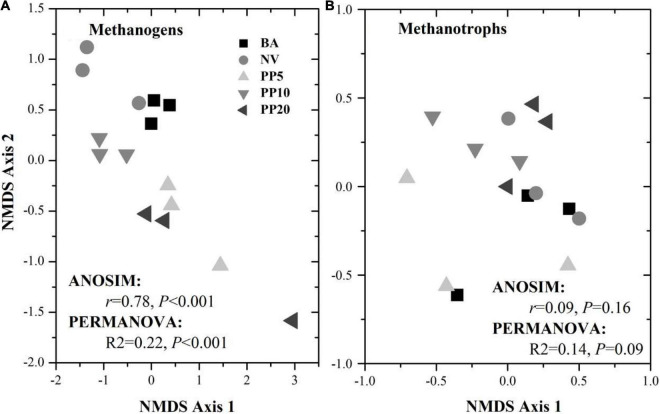
NMDS ordination for methanogenic **(A)** and methanotrophic **(B)** communities based on corresponding 16S rDNA sequences across different vegetation types. BA, bare land; NV, natural vegetation; PP5, 5-years old poplar plantation; PP10, the 10-year old poplar plantation; PP20, the 20-year old poplar plantation.

High throughput sequencing with primer set A533F/A934R obtained 166,465 sequences after quality filtering. Taxonomy classification identified a total of 96,178 archaeal sequences (5,371 - 7,827 sequences per sample) after deleting singletons. The data set was rarefied to 5,371 sequences per sample, and a total of 1810 OTUs existed in this rarefied OTU table. Then, 124 OTUs (18-867 sequences per samples) from 11 archaeal genera were identified as methanogen (Table 1). The alpha diversity indexes observed species was from 33 to 60 and chao1 from 40 to 84 across vegetation types, but both were not significant different across samples (*p* > 0.05; Supplementary Figure 2A). The methanogenic communities varied significantly across land types, confirmed by ANOSIM (*r* = 0.78, *p* < 0.001) and PERMANOVA (R^2^= 0.22, *p* < 0.001) (Figure 1A).

High throughput sequencing with primer set 343F/798R obtained 165,377 sequences after quality filtering. Taxonomy classification identified a total of 142,026 bacterial sequences (8,408 - 10,360 sequences per sample) after deleting singletons. The data set was rarefied to 8,408 sequences per sample, and a total of 9,770 OTUs existed in this rarefied OTU table. Then, 36 OTUs (0-46 sequences per samples) from 6 bacterial genera were identified as methanotrophic (Table 2). The alpha diversity indexes observed species was from 5 to 9 and chao1 from 9 to 11 across vegetation types, but both were not significant different across all samples (*p* > 0.05; Supplementary Figure 2B). The methanotrophic communities showed a homogeneous spread across land types (ANOSIM: *r* = 0.09, *p* = 0.16; PERMANOVA: *R*^2^= 0.14, *p* = 0.09; Figure 1B). Methanotrophs are divided into type-I (gammaproteobacteria) and type-II (alphaproteobacteria), and we found that most of the OTUs belonged to type-I methanotrophs, including *Crenothrix, Methylobacter, Methylomicrobium, Methylomonas and Methylosarcina.*

**TABLE 2:** Reads abundance of methanotrophic genera across different land types from 16S amplicon sequencing.

	BA	NV	PP5	PP10	PP20
*Crenothrix* (Type-I)	1	2	1	1	3
*Methylobacter* (Type-I)	1	0	0	1	1
*Methylomicrobium* (Type-I)	5	3	1	5	4
*Methylomonas* (Type-I)	2	8	2	9	17
*Methylosarcina* (Type-I)	6	9	9	2	4
Candidatus_*Methylacidiphilum*	1	0	0	0	0

*Data are mean reads abundance of samples per vegetation type (n = 3). BA, bare land; NV, natural vegetation; PP5, the 5-year old poplar plantation; PP10, the 10-year old poplar plantation; PP20, the 20-year old poplar plantation. Greyed cells indicate presence.*

### Abundance of *mcr*A and *pmo*A Genes

**FIGURE 2:**
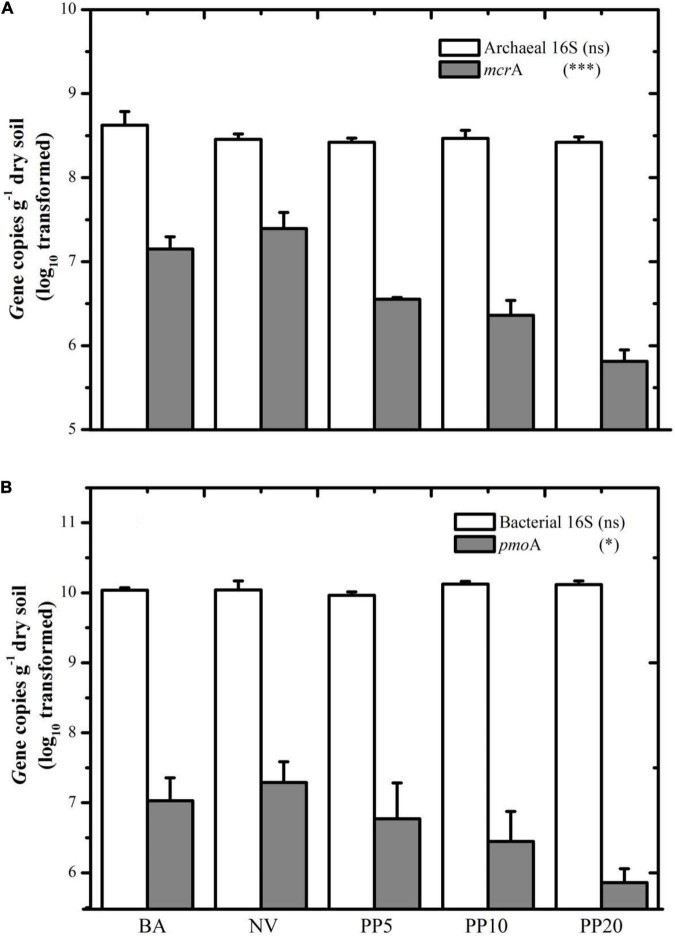
**(A)** Gene copy number of archaeal 16S and *mcr*A among different land types. **(B)** Gene copy number of Bacterial 16S and *pmo*A among different land types. BA: bare land; NV: natural vegetation; PP5: 5-years old poplar plantation; PP10: 10-years old poplar plantation; PP20: 20-years old poplar plantation. *p < 0.05; ^***^p < 0.001; ns: *p* > 0.05.

Archaeal and bacterial 16S rDNA abundances did not show significant differences across land types (*P* > 0.05), with archaeal abundance at ∼10^8^ copies g^–1^ dry soil (Figure 2A) and bacterial abundance at ∼10^10^ copies g^–1^ dry soil (Figure 2B) across all the land types.

Both *mcr*A and *pmo*A genes were present in all five land types (Figure 2). The *mcr*A gene copy number was lower in soils of PP5 (3.5 × 10^6^ copies/g dry soil), PP10 (2.3 × 10^6^ copies/g dry soil) and PP20 (6.4 × 10^5^ copies/g dry soil) than BA (1.4 × 10^7^copies/g dry soil) and NV (2.4 × 10^7^copies/g dry soil) (*p* < 0.001). Quantification of *the pmo*A gene showed a similar pattern to *the mcr*A gene, with lower copies in PP5, PP10, and PP20 (lower than 10^7^ copies/g dry soil) when compared to both un-afforested types (higher than 10^7^ copies/g dry soil) (*p* < 0.05).

### Deciphering Methane Metabolism With Metagenomics

**FIGURE 3:**
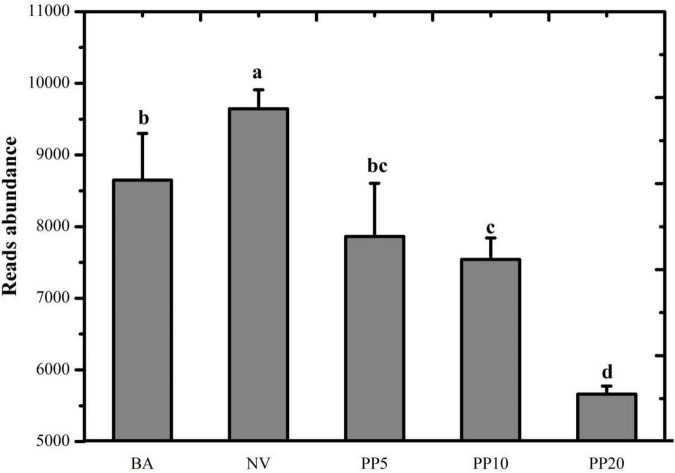
Read abundance of the KEGG pathway: ko00680 Methane metabolism in the soil of different land types. All reads belong to the category of methane metabolism. BA: bare land; NV: natural vegetation; PP5: 5-years old poplar plantation; PP10: the 10-year old poplar plantation; PP20: the 20-year old poplar plantation. Different letters mean significant difference as *p* < 0.05 in ANOVA.

A total of 526,742,669 reads (16,618,761 - 58,023,740 reads per sample) were achieved after quality filtering in the meta-genomic analysis. Read abundance of the ko00680 pathway showed significant differences across the five land types (*P* < 0.05; Figure 3) after normalization to one million reads per sample. NV obtained the highest read number. Reads abundance of the pathway in the PP10 and PP20 were significantly lower than those in both non-afforested BA and NV land types.

Two primary biochemical pathways of methanogenesis are CO_2_ to CH_4_ (M00567 KEGG Pathway) and acetate to CH_4_ (M00357 KEGG Pathway) (Supplementary Table 2). Methanogenesis through the acetoclastic pathway involves several enzymes such as acetate kinase, Acetyl-CoA decarboxylase, Acetyl-CoA synthetase, etc. Genes encoding these enzymes were shown significantly lower abundances in the three afforested land types than the two non-afforested types.

The *pmo*A-gene (EC:1.14.18.3, 1.14.99.39) which catalyze the oxidation of methane to methanol is widely used as a functional gene marker for methanotrophs. Abundances of the *pmo*A gene detected with metagenomic approach were higher in the PP10 and PP20 than the non-afforested BA and NV land types (Supplementary Table 3). The serine pathway (M00346 KEGG Pathway), the xylulose monophosphate pathway (M00344 KEGG Pathway) and the ribulose monophosphate pathway (M00345 KEGG Pathway) (Supplementary Table 3) are three formaldehyde assimilation pathways in the downstream of methane conversion to formaldehyde, which also are important biochemical pathways of methanotrophs (Bhattacharyya et al., 2017). The total read number of involved enzymes was significantly lower in the three afforested land types than the two non-afforested types for all the three pathways.

### *In situ* CH_4_ Flux at Sampling Event

**FIGURE 4:**
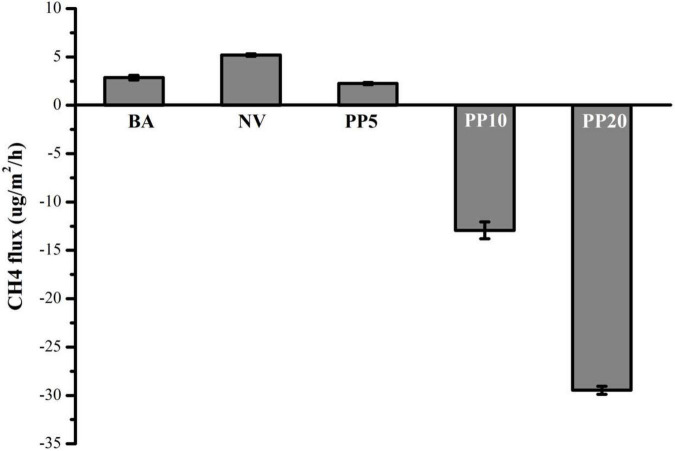
Net CH_4_ flux across five land types. Mean ± SEM (*n* = 4).

Afforestation decreased CH_4_ emissions compared to the BA and NV land types (One-way ANOVA: *F* = 2111, *p* < 0.001; Figure 4). In PP10 and PP20, the soils became a net CH_4_ sink (Figure 4).

### Random Forest Analysis

**FIGURE 5:**
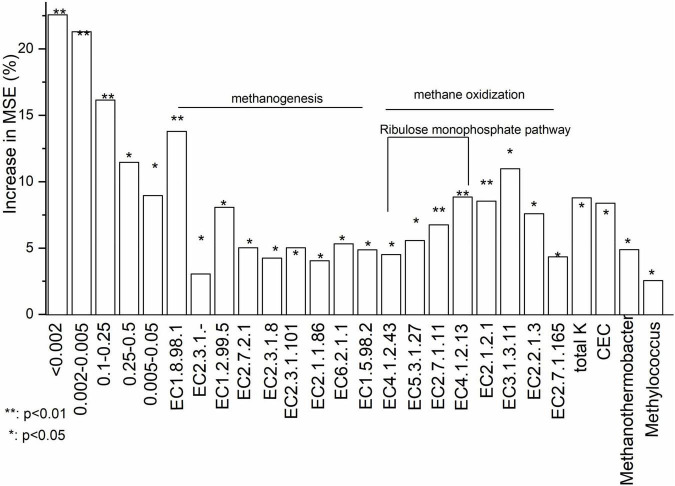
Relative importance of the abiotic and biotic factors in driving CH_4_ flux based on a Random Forest analysis. Increase in MSE (%) represented ‘Increased in mean squared error (%)‘ which means the contribution of this independent variable to the prediction accuracy of the dependent variable. Higher Increase in MSE (%) value means higher importance of this independent variable. EC 1.8.98.1: Heterodisulfide reductase; EC 2.3.1.-: Acetyl-CoA decarboxylase; EC 1.2.99.5: Formylmethanofuran dehydrogenase; EC 2.7.2.1: Acetate kinase; EC 2.3.1.8: Phosphate acetyltransferase; EC 2.3.1.101: Formylmethanofuran tetrahydromethanopterin-N-formyltransferase; EC 2.1.1.86: Tetrahydromethanopterin-S-methyltransferase (A-H); EC 6.2.1.1: Acetyl-CoA synthetase; EC 1.5.98.2: 5,10-methylenetetrahydromethanopterin reductase; EC 4.1.2.43: 3-hexose-6-phosphate synthase; EC 5.3.1.27: 6-phospho-3-hexoisomerase; EC 2.7.1.11: 6-phophofructokinase; EC 4.1.2.13: Fructose bisphosphate aldolase; EC 2.1.2.1: Glycine hydroxymethyl transferase; EC 3.1.3.11: Fructose bisphosphate; EC 2.2.1.3: Formaldehyde transketolase; EC 2.7.1.165: Glycerate-2-kinase.

Basing on Increase in MSE (%) value, random forest analysis identified that soil particle size composition and functional genes for key enzymes were most important in determining CH_4_ flux (%) (Figure 5). Increase in MSE (%) represented ‘Increased in mean squared error (%)’ which means the contribution of this independent variable to the prediction accuracy of the dependent variable. Higher Increase in MSE (%) value means higher importance of this independent variable. Soil dry matter and oxygen concentration were not significant in explaining CH_4_ fluxes. With random forest analysis, five soil particle size components, nine genes involved in methanogenesis and eight genes involved in methane oxidation were shown to be significant for variations in CH_4_ fluxes (Figure 5). Total K and CEC were significant soil chemical characteristics for variations in CH_4_ fluxes. *Methanothermobacter* and *Methylococcus* were identified, respectively, as methanogenic and methanotrophic genera in explaining variations in CH_4_ fluxes. Data of all soil properties were listed in Supplementary Table 4.

### Structural Equation Modeling

**FIGURE 6:**
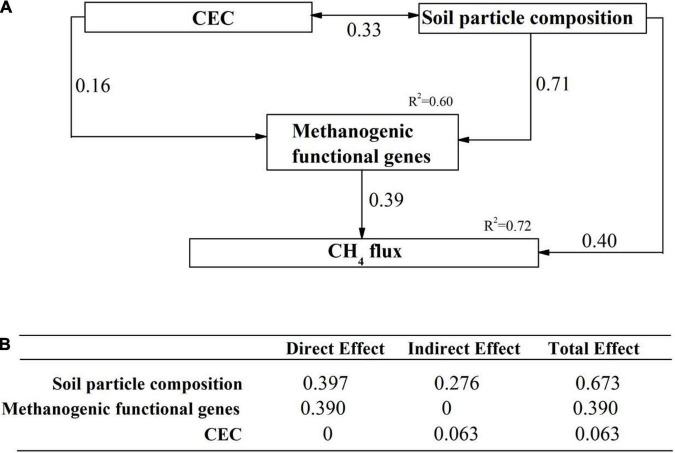
The direct and indirect effects of abiotic and biotic factors on CH_4_ flux. **(A)** A structural equation model showing the standardized total effects of soil properties and methanotrophs on CH_4_ flux; **(B)** The standardized direct and indirect effects of factors mentioned above on CH_4_ flux. The numbers above the arrows represented path coefficients. CEC: Cation exchange capacity; methanogenic functional genes: metagenome-based methanogenic gene frequency identified to be important predictors in random forest analysis; soil particle composition: soil particles identified to be important predictors in random forest analysis.

The direct and indirect effects of soil properties, microbial communities and functional genes on CH_4_ flux was explored using SEM analysis. Our SEM model adequately fitted the data (Chi-square = 1.249, df = 1, Probability level = 0.264, RSMEA = 0.001, TLI = 1.000) and explained 72% variation of CH_4_ flux (Figure 6). Soil particle composition, the functional genes related to methanogenesis and CEC were included in the SEM model. Soil particle size and the methanogenic functional genes comprised the two dominant direct effects on CH_4_ flux (Figure 6). Soil particle size had the highest total effect on CH_4_ flux, with indirect effects by methanogenic functional genes and CEC. Based on the variables selected from random forest analysis, we constructed several hypothetical models before performing SEM analysis. Any SEM model including methanotrophs can not fit the statistical requirements, e.g., RMSEA < 0.05 and TLI > 0.95.

## Discussion

### Drivers of CH_4_ Flux

Water-saturated, carbon-rich ecosystems such as wetlands are traditionally classified as net CH_4_ sources (Tan et al., 2015). Here this study showed that afforestation decreased CH_4_ emissions compared to the natural marshland, and the soils appeared to become net CH_4_ sinks 10 years after afforestation. This suggests that afforestation on the degraded marshlands of the Yangtze River could help reduce CH_4_ emissions. Such was also observed in the conversion to forest from grasslands (Benanti et al., 2014) and croplands (Wu et al., 2018).

Methane emission from the soil is driven by the balance between microbial CH_4_ production and CH_4_ consumption, together with soil physicochemical properties that further affect CH_4_-metabolizing microbes and CH_4_ diffusion (Conrad, 2002). Studies have attempted to disentangle the various drivers of methane fluxes, such as temperature, moisture, microbial diversity and the abundance of CH_4_-metabolizing microbial populations (St Pierre et al., 2019; Lang et al., 2020). Such studies have focused on the correlation between CH_4_ emission and a specific variable. However, the relative importance of such factors was never determined within a comprehensive framework. The primary purpose of our study was to simultaneously consider interactions of abiotic and biotic factors in order to investigate the interplay between methane-cycling microbial communities, functional metabolic pathways and soil properties as they affect CH_4_ flux in various land types.

By combining random forest analysis and structural equation modeling, our results provide novel evidence of a strong correlation between the CH_4_ flux and the abundance of functional genes related to both CH_4_ production and CH_4_ consumption. Many studies suggest a coupling between microbial community structure and methane production or consumption (e.g., Subhajit et al., 2018; Yu et al., 2020). However, some previous studies suggest that coarse measures of microbial communities based on DNA techniques may be insufficient to understand the functional contributions of such microbial communities (Rocca et al., 2015; Wood et al., 2015). A few examples illustrate this point. First, metaproteomics revealed that perturbations in organic matter affected functional composition over the taxonomic composition of the microbial community (Mikan et al., 2020). Second, grazing clearly changed the overall microbial functional composition, but the overall composition of the CH_4_-cycling microbial community was unchanged (Ma et al., 2019). Third, some studies have shown that considerable variation in atmospheric CH_4_ consumption could occur without apparent changes in biomass and composition of the high-affinity methanotrophic community (e.g., Menyailo et al., 2010). Our results also demonstrate that variation in functional gene composition due to land type was more likely responsible for the observed effects on CH_4_ flux than shifts in taxonomic microbial community structure. One possible explanation for the poor linkage between taxonomic microbial community structure and microbial community function may be sufficient functional redundancy (Paul, 2007) among microbial taxa to obscure the linkage. To our knowledge, this is the first report providing evidence consistent with the hypothesis that functionality is superior to taxonomic community structure in determining *in situ* CH_4_ flux.

Several functional genes involved in methanogenic pathways showed strong explanatory strength in CH_4_ flux (Supplementary Table 2). This was supported by two recent studies which reported metagenomic genes were predictive for CH_4_ flux in industrial salt ponds (Zhou et al., 2022) and arctic lake sediment (Emerson et al., 2021). Several genes such as genes for EC 2.1.1.86 and EC 2.7.2.1 were identified for significantly correlated with CH_4_ flux in our study and a previous study (Zhou et al., 2022). The enzyme EC 2.1.1.86 represents tetrahydromethanopterin S-methyltransferase for generation of methyl-CoM, the next to last step in hydrogenotrophic and acetoclastic methanogenesis. The EC 2.7.2.1 metabolize acetate into acetyl-CoA for methanogenesis. However, different from that *mcr*A gene was predictive for CH_4_ flux (Emerson et al., 2021; Zhou et al., 2022), *mcr*A gene wasn’t found to be significant in explaining CH_4_ flux in our study. This suggested that the limiting step of CH_4_ production in our study sites were in the upstream steps (e.g., biochemical reaction was catalyzed by EC 2.1.1.86 and EC 2.7.2.1) but not in the terminal step in methanogenesis catalyzed by methyl coenzyme M reductase (*mcr*A gene).

There existed a strong interactive effect of functional genes and soil properties on CH_4_ flux. Soil particle size and CEC were the two soil property drivers. Soil particles < 0.002 mm were particularly important in affecting CH_4_ flux, as shown in random forest analysis. This was supported by a previous study showing that methane production was at a higher rate in clay (size < 0.002 mm) than in coarser particles (e.g., sand, gravel) (Wagner et al., 1999). Small soil particles enhance water retention, promote higher microbial biomass (Wilpiszeski et al., 2019), and reduced oxygen permeability, leading to lower redox potential and even local anoxia. This, in turns, supports higher CH_4_ production and suppresses CH_4_ oxidation. Our structural equation modeling also showed that soil particle size could interact with CEC to affect CH_4_ production, supported by a previous study (Mitra et al., 2002). Clay and humus particles are negatively charged, which significantly influence CEC. CEC was higher in clay than in gravel or sand (Wagner et al., 1999). van Loosdrecht et al. (1987a,b) showed that the hydrophobicity and electrophoretic mobility of microbes could be taken as an indicator for their adhesion properties. Methanogens, e.g., *Methanosarcina barkeri*, have a hydrophobic cell surface and low electrophoretic mobility, which increases their attachment to soil particles (Grotenhuis et al., 1992).

### Community and Abundance of Methanotrophs

Overall, the motivation of this study was not to describe the structures of communities of methanogens and methanotrophs, but result of the methanotrophic community is discussed here because it is inconsistent with our hypothesis.

Copy number of the *pmo*A gene did not support our hypothesis that afforestation would support growth of the population of methanotrophs; *pmo*A gene copy number decreased in the afforested plots. In contrast with previous results showing methanotrophic population recovery with plantation age (Nazaries et al., 2011; Sun and Badgley, 2019), our study showed that the metabolic pathway of microbial methanotrophic activity was suppressed with increasing plantation age. These results can probably be best explained by the significant decrease in methanogenic activity with plantation age, as evidenced by the decreasing *mcr*A gene and methanogenesis pathway abundance in older plantations and the shift to becoming a net CH_4_ sink.

Our study also showed that afforestation in the Yangtze River marshland did not change the community structure of aerobic bacterial methanotrophs. This, again, is different from previous studies showing a shift in methanotrophic communities following afforestation (Singh et al., 2007, 2009; Nazaries et al., 2011). Previous studies showed that afforestation on pasture or reforestation increased the abundance of type-II methanotrophs but suppressed type-I methanotroph (Nazaries et al., 2011; Sun and Badgley, 2019). It was also found that type-II methanotrophs were dominant (77% of methanotrophs) and mainly drove the changes in methane monooxygenase gene abundances as plantations aged (Sun and Badgley, 2019). Here, bacterial community composition showed that type I methanotroph was dominant, including *Methylobacter*, *Methylomicrobium*, *Methylomonas*, and *Methylosarcina*. A reasonable explanation for our observations is that our site undergoes periodical flooding and produces high amounts of methane irrespective of plantation age, in contrast to the studies mentioned above. As methanotrophs are aerobic, soil water content may be an important consideration that overrides afforestation as a controlling factor of methanotrophic communities in marshlands (Zhang et al., 2019). This is supported by previous studies that show CH_4_ oxidation rates decrease in temperate forest when soil water content ranges from 60 to 100% water-filled pore space (Whalen and Reeburgh, 1990; Bender and Conrad, 1995).

### Implications, Limitations and Future Directions

The different molecular methods used here (amplicon sequencing, qPCR, and metagenomics) each have their strengths and weaknesses and were used to complement each other. For example, amplicon sequencing provides an excellent taxonomic overview for many samples and allows detecting even very rare members of the community, but is limited to one gene at a time. Metagenomics, on the other hand, enables studying community functions in an untargeted way. However, both methods deliver only compositional data with many limitations. qPCR, therefore, complements these methods by providing absolute gene quantity values. A significant advantage of studying methanogens and methanotrophs, in contrast to some other microbial guilds, is that each is limited to a single energy-yielding process for growth. Therefore, the population sizes of these two guilds serve as a good proxy for the magnitude of gross methane production and consumption. A variety of other studies have demonstrated correlations between methane production and soil properties as well as microbiome features (Emerson et al., 2021; Zhou et al., 2022). Here, our study revealed the relative importance and interactive pathway of soil properties and microbial features in explaining *in-situ* CH_4_ flux including various molecular methods into analysis. Thus, we think our findings could, to some extent, provide some new insights into driving mechanism of CH_4_ flux and cycling. This suggests that functional gene data could be used to predict methane fluxes by advanced modeling. It is important to recognize that, in general, the assumption about microbe — methane flux interaction has been formulated for several times from 2,000 until to date based on limited datasets (Täumer et al., 2021). Here our data set is also limited to make a conclusion that how it can be done. Even so, we still think our findings could at least provide some new insights into driving mechanism of CH_4_ flux and cycling in the studied ecosystem, i.e., gene abundances obtained through metagenomics or quantitative/digital PCR could be more effective than community profiling in predicting CH_4_ fluxes. Further, although qPCR abundance of pmoA was previously significantly correlated to methane oxidation activity of methanotrophs in forest soils (Kolb et al., 2005), we here found no relationship between both. Our results showed high abundances of methanotrophs present according to the qPCR data but low abundances of reads that encode for the *pmo*A in metagenomic analysis (as listed in Supplementary Table 3). This might be originated from rarefaction of abundance data. Rarefaction was done to avoid bias in sequencing depth. Prior to rarefaction, abundances of *pmo*A gene in metagenomics analysis were from 25 to 173. Basically, our qPCR data showed that ratio of *pmo*A gene to bacterial 16S rDNA was about 0.01% ∼ 0.1% (10^6^ ∼ 10^7^/10^10^), meaning that in 10^7^ metagenome reads ca. 100 – 1,000 will be affiliated with a methanotroph. However, that is all the genes, finding a *pmo*A is still rare. As annotated reads in our metagenomic analysis was at 10^7^ level, we think data of *pmo*A gene in metagenomic after rarefaction was reasonable, and results of meta-genome and qPCR approaches could support each other.

Our study provides empirical evidence that microbial community function is more important than taxonomic community structure in explaining *in situ* CH_4_ fluxes. We first highlight an interactive effect between methanogenic functional genes and soil properties, in particular soil particle size. The results should urge researchers to adopt the use of functional gene analysis through metagenomics to develop a gene-centric approach and to utilize a framework that includes known interactions among factors that can be integrated into simulation models to understand climate change and to inform management policies. One of the key limitations of this study is that sampling was done at only one time on one tree species with only three replicates for each vegetation type, which limited us to make a final conclusion about basic scientific assumptions on microbe-methane flux interaction. Furthermore, besides community composition and functional gene, the activity of all microbes engaged in methane-cycle is also important. Future study should consider to include meta-transcriptome and meta-proteome revealling *in-situ* microbail activities in prediction of CH_4_ flux. Third, some soil characteristics such as soil temperature was not recorded here which was thought to be important for CH_4_ flux and should be included into analysis in future study. Therefore, future work should take more environmental variables and methodological approach into account and include larger temporal and spatial scales across a variety of land use types and climates.

## Data Availability Statement

The datasets presented in this study can be found in online repositories. The names of the repository/repositories and accession number(s) can be found here https://www.ncbi.nlm.nih.gov/sra/PRJNA787026.

## Author Contributions

QZ: conceptualization, data curation, formal analysis, writing—original draft, and writing—review and editing. JT: methodology and writing—original draft. RA: writing—review and editing. DW: methodology, formal analysis, and writing—review and editing. XH: methodology, writing—original draft, and writing—review and editing. SG: methodology, data curation, and formal analysis. LZ: methodology, data curation, formal analysis, and writing—original draft. YT: re-sources, methodology, and writing—review and editing. XZ: re-sources, methodology, and writing—review and editing. HY: writing—original draft, funding acquisition, and writing—review and editing. QS: conceptualization, methodology, writing—original draft, and funding acquisition. All authors contributed to the article and approved the submitted version.

## Conflict of Interest

The authors declare that the research was conducted in the absence of any commercial or financial relationships that could be construed as a potential conflict of interest.

## Publisher’s Note

All claims expressed in this article are solely those of the authors and do not necessarily represent those of their affiliated organizations, or those of the publisher, the editors and the reviewers. Any product that may be evaluated in this article, or claim that may be made by its manufacturer, is not guaranteed or endorsed by the publisher.

## Footnotes

1
http://ccb.jhu.edu/software/FLASH/

2
http://drive5.com/uparse/

3
http://sourceforge.net/projects/rdp-classifier/

4
https://greengenes.lbl.gov/Download/Taxonomic_Outlines/

5
https://www.ncbi.nlm.nih.gov/Taxonomy/taxonomyhome.html/index.cgi?chapter~=~tgencodes#SG1

6
http://www.bioinformatics.org/cd-hit/

7
http://www.kegg.jp

8
http://cran.rproject.org/web/packages/rfPermute/rfPermute.pdf
