# Supplementary material for: Soil Properties Interacting With Microbial Metagenome in Decreasing CH4 Emission From Seasonally Flooded Marshland Following Different Stages of Afforestation
Source: Front Microbiol. 2022 Feb 23;13:830019. doi: 10.3389/fmicb.2022.830019 (PMC8905362; doi:10.3389/fmicb.2022.830019)
Supplement: Supplementary file 2 [file Data_Sheet_1.docx]

**Table S1** Primers used to amplify the taxonomic and functional genes

| Primer | Target | Sequence (5`-3`） | Assay |  |
| --- | --- | --- | --- | --- |
| 343F | Bacterial 16S | TACGGRAGGCAGCAG | Miseq | Du et al., 2019 |
| 798R |  | AGGGTATCTAATCCT |  |  |
| A533F | Archaeal 16S | TGCCAGCCGCCGCGGTAA | Miseq | Brunk and Eis. 1998 |
| A934R |  | GTGCTCCCCCGCCAATTCCT |  | Großkopf et al., 1998 |
| Ba519F | Bacterial 16S | CAGCMGCCGCGGTAANWC | qPCR | Stubner, 2002 |
| Ba907R |  | CCGTCAATTCMTTTRAGTT |  |  |
| Ar364F | Archaeal 16S | CGGGGYGCASCAGGGGCGAA | qPCR | Burggraf et al.,1997 |
| Ar934R |  | GTGCTCCCCCGCCAATTCCT |  | Großkopf et al., 1998 |
| mlasmod - F | *mcr*A | GGYGGTGTMGGDTTCACMCARTA | qPCR | Angel et al.,2012 |
| mcrA-rev - R |  | CGTTCATBGCGTAGTTVGGRTAGT |  |  |
| A189f | *pmo*A | GGNGACTGGGACTTCTGG | qPCR | Costello and Lidstrom 1999 |
| mb661r |  | CCGGMGCAACGTCYTTACC |  |  |

Angel R, Claus P, Conrad R. Methanogenic archaea are globally ubiquitous in aerated soils and become active under wet anoxic conditions.*ISME J* 2012; 6: 847–862.

Brunk CF, Eis N. Quantitative measure of small subunit rRNA gene sequences of the Kingdom Korarchaeota. *Appl Environ Microbiol*1998; 64(12): 5064-5066.

Burggraf S, Huber H, Stetter KO. Reclassification of the crenarchaeal orders and families in accordance with 16S rRNA sequence data. *Int J Syst Evol Microbiol* 1997; 47: 657–660.

Costello AM, Lidstrom ME. Molecular characterization of functional and phylogenetic genes from natural populations of methanotrophs in lake sediments. *Appl Environ Microbiol* 1999; 65: 5066-5074.

Du F, Li Y, Tang Y, Su S, Yu J, Yu F, Li J, Li H, Wang M, Xu P. Response of the gut microbiome of *Megalobrama amblycephala* to crowding stress. *Aquaculture*2019; 500: 586-596.

Großkopf R, Janssen PH, Liesack W. Diversity and structure of the methanogenic community in anoxic rice paddy soil microcosms as examined by cultivation and direct 16S rRNA gene sequence retrieval.*Appl Environ Microbiol* 1998; 64: 960–969.

Stubner S. Enumeration of 16S rDNA of Desulfotomaculum lineage 1 in rice field soil by real-time PCR with SybrGreen™ detection.*J Microbiol Methods* 2002; 50: 155–164.

**Table S2**Abundance of reads that encode for enzymes involved in methanogenesis

| **Methanogenesis pathway** | **Enzyme function** | **BA** | **NV** | **PP5** | **PP10** | **PP20** |
| --- | --- | --- | --- | --- | --- | --- |
| Carbon dioxide (CO_2_) to methane (CH_4_) production  (M00567 KEGG Pathway) | Formylmethanofuran dehydrogenase [EC1.2.99.5] | 104 | 102 | 86 | 83 | 69 |
|  | Formylmethanofuran tetrahydromethanopterin-N-formyltransferase  [EC 2.3.1.101] | 22 | 24 | 19 | 18 | 16 |
|  | Methenyltetrahydromethanopterin cyclohydrolase [EC 3.5.4.27] | 21 | 24 | 18 | 18 | 15 |
|  | Methylenetetrahydromethanopterin dehydrogenase [EC 1.5.98.1] | 0.15 | 0 | 0 | 0 | 0 |
|  | 5,10-methenyltetrahydromethanopterin hydrogenase [EC 1.12.98.2] | 0 | 0 | 0 | 0 | 0 |
|  | 5,10-methylenetetrahydromethanopterin reductase [EC 1.5.98.2] | 113 | 130 | 97 | 95 | 76 |
|  | Tetrahydromethanopterin-S-methyltransferase (A-H) [EC 2.1.1.86] | 4 | 3 | 1 | 1 | 0.43 |
|  | Methyl coenzyme M reductase [EC 2.8.4.1] | 0.42 | 0.16 | 0.05 | 0.04 | 0 |
|  | Heterodisulﬁde reductase [EC 1.8.98.1] | 162 | 152 | 123 | 109 | 67 |
|  | **Total abundance** | **426.57** | **435.16** | **344.05** | **324.04** | **243.43** |
| Acetate to Methane (CH_4_  ) production  (M00357 KEGG Pathway) | Acetate kinase [EC 2.7.2.1] | 178 | 216 | 164 | 157 | 87 |
|  | Phosphate acetyltransferase [EC 2.3.1.8] | 247 | 298 | 230 | 221 | 151 |
|  | Acetyl-CoA synthetase [EC 6.2.1.1] | 806 | 902 | 757 | 711 | 517 |
|  | Acetyl-CoA decarboxylase [EC 2.3.1.-] | 1037 | 1271 | 941 | 895 | 620 |
|  | Tetrahydromethanopterin-S-methyltransferase (A–H)  [EC 2.1.1.86] | 4 | 3 | 1 | 1 | 0.43 |
|  | Methyl coenzyme M reductase [EC 2.8.4.1] | 0.42 | 0.16 | 0.05 | 0.04 | 0 |
|  | Heterodisulﬁde reductase [EC 1.8.98.1] | 162 | 152 | 123 | 109 | 67 |
|  | **Total abundance** | **2434.42** | **2842.16** | **2216.05** | **2094.04** | **1442.43** |

**Table S3**Abundance of reads that encode for enzymes involved in methanotrophic pathway

| **Methanotrophic pathway** | **Enzyme function** | **BA** | **NV** | **PP5** | **PP10** | **PP20** |
| --- | --- | --- | --- | --- | --- | --- |
| Methane oxidation | Particulate methane monooxygenase (EC 1.14.18.3) | 1.8 | 1.3 | 1.7 | 2.2 | 3.4 |
| Serine pathway  (M00346 KEGG Pathway) | Glycine hydroxymethyl transferase [EC 2.1.2.1] | 278 | 312 | 252 | 233 | 171 |
|  | Serine glyoxylate transaminase [EC 2.6.1.45] | 241 | 270 | 216 | 211 | 167 |
|  | Glycerate dehydrogenase [EC 1.1.1.29] | 20 | 22 | 19 | 19 | 14 |
|  | Glycerate-2-kinase [EC 2.7.1.165] | 8 | 9 | 7 | 6 | 4 |
|  | Phosphopyruvate hydratase [EC 4.2.1.11] | 262 | 298 | 247 | 241 | 174 |
|  | Phosphoenol pyruvate carboxylase [EC 4.1.1.31] | 182 | 222 | 160 | 169 | 128 |
|  | Malate dehydrogenase [EC 1.1.1.37] | 288 | 326 | 273 | 266 | 192 |
|  | Malate CoA ligase [EC 6.2.1.9] | 11 | 13 | 9 | 10 | 11 |
|  | Malyl CoA Lyase [EC 4.1.3.24] | 32 | 42 | 29 | 29 | 23 |
|  | **Total abundance** | **1322** | **1514** | **1212** | **1184** | **884** |
| Ribulose monophosphate pathway  (M00345 KEGG Pathway) | 3-hexose-6-phosphate synthase [EC 4.1.2.43] | 15 | 19 | 12 | 11 | 8 |
|  | 6-phospho-3-hexoisomerase [EC 5.3.1.27] | 14 | 16 | 11 | 10 | 6 |
|  | 6-phophofructokinase [EC 2.7.1.11] | 387 | 434 | 368 | 330 | 228 |
|  | Fructose bisphosphate aldolase [EC 4.1.2.13] | 223 | 231 | 197 | 183 | 143 |
|  | **Total abundance** | **639** | **700** | **588** | **534** | **385** |
| Xylulose monophosphate pathway  (M00344 KEGG Pathway) | Formaldehyde transketolase [EC 2.2.1.3] | 12 | 14 | 12 | 10 | 7 |
|  | Glycerone kinase [EC 2.7.1.29] | 17 | 17 | 18 | 15 | 16 |
|  | Fructose bisphosphate aldolase [EC 4.1.2.13] | 223 | 231 | 197 | 183 | 143 |
|  | Fructose bisphosphate [EC 3.1.3.11] | 194 | 208 | 179 | 160 | 113 |
|  | **Total abundance** | **446** | **470** | **406** | **368** | **279** |

**Fig.S1** Sampling site distribution along the Yangtze river.


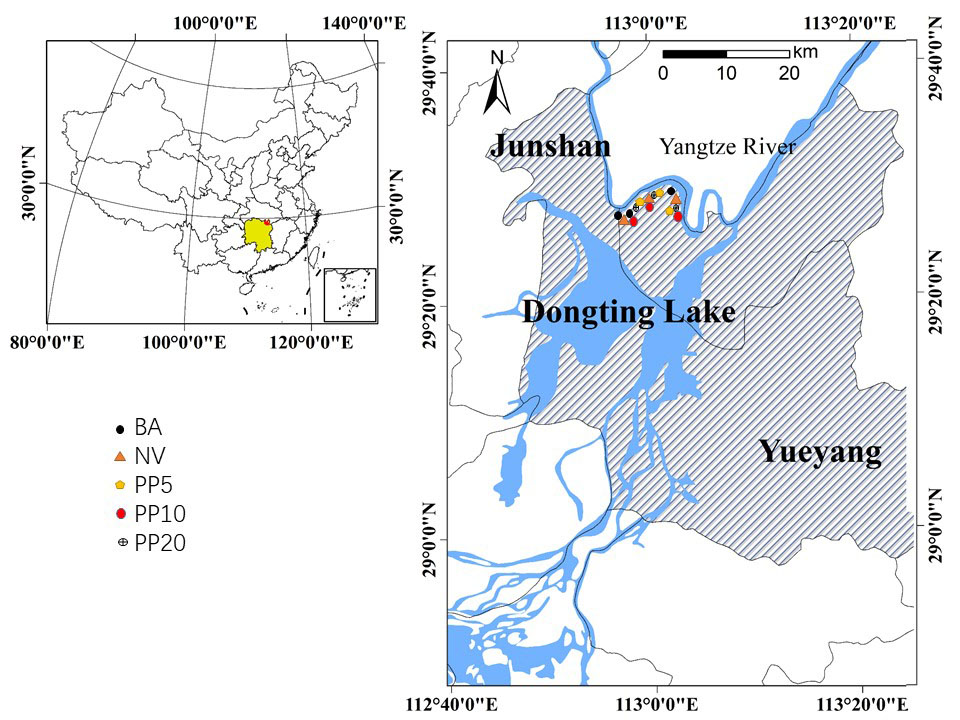


**Fig S2** Two alpha diversity indexes, observed species and chao1, calculated basing on 16S amplicon sequencing for methanogens (a) and methanotrophs (b)
